# Stakeholder perspectives on the implementation and impact of Indigenous health interventions: A systematic review of qualitative studies

**DOI:** 10.1111/hex.13230

**Published:** 2021-03-17

**Authors:** Shingisai Chando, Allison Tong, Martin Howell, Michelle Dickson, Jonathan C. Craig, Jack DeLacy, Sandra J. Eades, Kirsten Howard

**Affiliations:** ^1^ Sydney School of Public Health The University of Sydney Sydney NSW Australia; ^2^ Centre for Kidney Research The Children's Hospital at Westmead Westmead NSW Australia; ^3^ College of Medicine and Public Health Flinders University Adelaide SA Australia; ^4^ Medical School Curtin University Perth WA Australia

**Keywords:** health policy, health services, impact evaluation, Indigenous health, outcomes

## Abstract

**Background:**

Evaluations of health interventions for Indigenous peoples rarely report outcomes that reflect participant and community perspectives of their experiences. Inclusion of such data may provide a fuller picture of the impact of health programmes and improve the usefulness of evaluation assessments.

**Aim:**

To describe stakeholder perspectives and experiences of the implementation and impact of Indigenous health programmes.

**Methods:**

We conducted a systematic review of qualitative studies evaluating complex health interventions designed for Indigenous communities in high‐income countries. We searched 6 electronic databases (through to January 2020): MEDLINE, PreMEDLINE, Embase, PsycINFO, EconLit and CINAHL and hand‐searched reference lists of relevant articles.

**Results:**

From 28 studies involving 677 stakeholders (mostly clinical staff and participants), six main themes were identified: enabling engagement, regaining control of health, improving social health and belonging, preserving community and culture, cultivating hope for a better life, and threats to long‐term programme viability.

**Conclusion:**

The prominence of social, emotional and spiritual well‐being as important aspects of the health journey for participants in this review highlights the need to reframe evaluations of health programmes implemented in Indigenous communities away from assessments that focus on commonly used biomedical measures. Evaluators, in consultation with the community, should consistently assess the capacity of health professionals to meet community needs and expectations throughout the life of the programme. Evaluations that include qualitative data on participant and community‐level outcomes can improve decision‐makers' understanding of the impact that health programmes have on communities.

**Patient or public contribution:**

This paper is a review of evaluation studies and did not involve patients or the public.

## 1 BACKGROUND

Evaluations of health programmes designed for Indigenous people are not always considered useful when making decisions about the development or future of health programmes, constituting a significant waste in resources.^1^, ^2^, ^3^ The focus on Western biomedical measures often means evaluations exclude outcomes that reflect participant experiences providing an incomplete picture of processes that are working well and those that are failing.^1^, ^4^ Increasingly, evaluations are using qualitative methods to capture participant experiences; however, this is often an afterthought or to support quantitative evaluation data and little attention is given to the importance of qualitative data. As qualitative evaluation data on the experiences of Indigenous people with health services grows, it is clear that these data should be given prominence and should be routinely collected and reported in evaluations of health programmes to ensure decision making reflects the needs of Indigenous communities.^1^

A fundamental challenge when conducting a programme evaluation is determining, and then measuring, the most appropriate outcomes to accurately reflect the scope of value and benefits of health programmes for individual participants and communities.^2^, ^5^ The outcomes considered in health evaluations are limited by the perspective of those commissioning the evaluation, cost and the availability and accessibility of the data needed for the report. As such, evaluations often assess measurable clinical outcomes.^1^, ^4^ However, unlike clinical outcomes, information about how Indigenous participants experience health programmes may help to identify the specific aspects of service delivery influencing service use or engagement which is essential to achieving long‐term health outcomes.^6^

A recent systematic review of Indigenous health evaluations outlined experiential outcomes, not typically measured and reported such as trust and empowerment.^4^ It is well established that political disenfranchisement, social and economic disadvantage and discrimination^7^ have eroded trust of government institutions for Indigenous communities in high‐income countries and^8^, ^9^ often excluded them when developing interventions to improve their health and quality of life.^8^ Previous research has demonstrated that services which fail to implement processes that secure the trust of the Indigenous communities and empower people to achieve their health goals, fail to sustain engagement.^6^ However, little is known about how widespread these experiential outcomes are in Indigenous communities to warrant consistent inclusion in evaluation reports and equally important, the facilitators and barriers to engagement with health services and ultimately the achievement of long‐term health outcomes.^10^

This systematic review aims to describe stakeholder (programme participant and community) perspectives and experiences with the implementation and impact of Indigenous health programmes, outlining the experiential outcomes, the reasons they are reported by respondents and the specific aspects of service delivery that influences achieving them. We acknowledge that the term ‘Indigenous’ is inadequate in representing the heterogeneity of the cultures and traditions of the populations described in this paper. We recognize that a more acceptable global collective term is needed.

## 2 METHODS

We followed the Enhancing Transparency of Reporting the Synthesis of Qualitative Research (ENTREQ) framework.^11^

### 2.1 Inclusion criteria

Eligible studies included primary studies, published in peer‐reviewed journals where the main objective was to evaluate a complex health intervention developed specifically for an Indigenous community. We defined complex interventions as interventions with several interacting components bringing together multiple systems and stakeholders to achieve programme delivery with multiple and multi‐level outcomes versus the ‘simple’ interventions which focus on individual‐level outcomes.^12^ We included evaluations conducted after a health intervention had been developed and implemented (e.g. process evaluations, outcome evaluations and economic evaluations), as defined by the Centers for Disease Control. We excluded formative evaluations because they are part of the programme development process and we were interested in the benefits of health interventions to Indigenous communities once fully implemented.^13^ Studies that reported qualitative data on the experiences and perspectives of stakeholders on the implementation and impact of Indigenous health interventions were eligible. Stakeholders included Indigenous participants in the programmes, family, staff and community members with knowledge about the health programme. Only studies of interventions among Indigenous communities from countries classified as ‘high income’ by the World Bank in 2017^14^ were included because of the similarities in the health inequities they experience compared to the non‐Indigenous populations within the same countries. Non‐English studies were excluded due to lack of resources for translation.

### 2.2 Data sources and searches

The search strategy is provided in Table S1. We conducted searches in MEDLINE, PreMEDLINE, Embase, PsycINFO, EconLit and CINAHL from inception to January 2020. Reference lists of relevant systematic reviews and included studies were searched for additional studies. Title and abstract screening were undertaken by SC to identify articles for full‐text screening. An independent title and abstract screen against the inclusion criteria were undertaken by MH. Disagreements were resolved through discussion and consultation with KH. Full texts of the remaining articles were assessed for study eligibility.

### 2.3 Appraisal of reporting

We critically appraised the quality of reporting for included papers using the Consolidated Criteria for Reporting Qualitative Research (COREQ).^15^ This framework included criteria specific to the research team, study methods, context of the study, analysis and interpretations. The studies were independently assessed by authors SC and JD. Disagreements were resolved through discussion.

### 2.4 Data analysis

We used thematic analysis to analyse data from the qualitative studies following guidelines from Thomas and Harden.^16^ Preliminary themes were developed and refined initially through triangulation among SC, AT and MD, and the final set of preliminary themes and subthemes was approved by the whole author group. Preliminary themes were uploaded into NVivo version 12^17^ along with all stakeholder quotations and text from the ‘results/findings’ and ‘discussion/conclusion’ sections of the included papers. SC conducted line‐by‐line coding of each study, inductively identifying concepts about stakeholder perspectives on participant experiences with and the impact of programmes. A thematic schema was developed through discussion with all authors to depict conceptual patterns and links among themes.

## 3 RESULTS

### 3.1 Literature search

**FIGURE 1:**
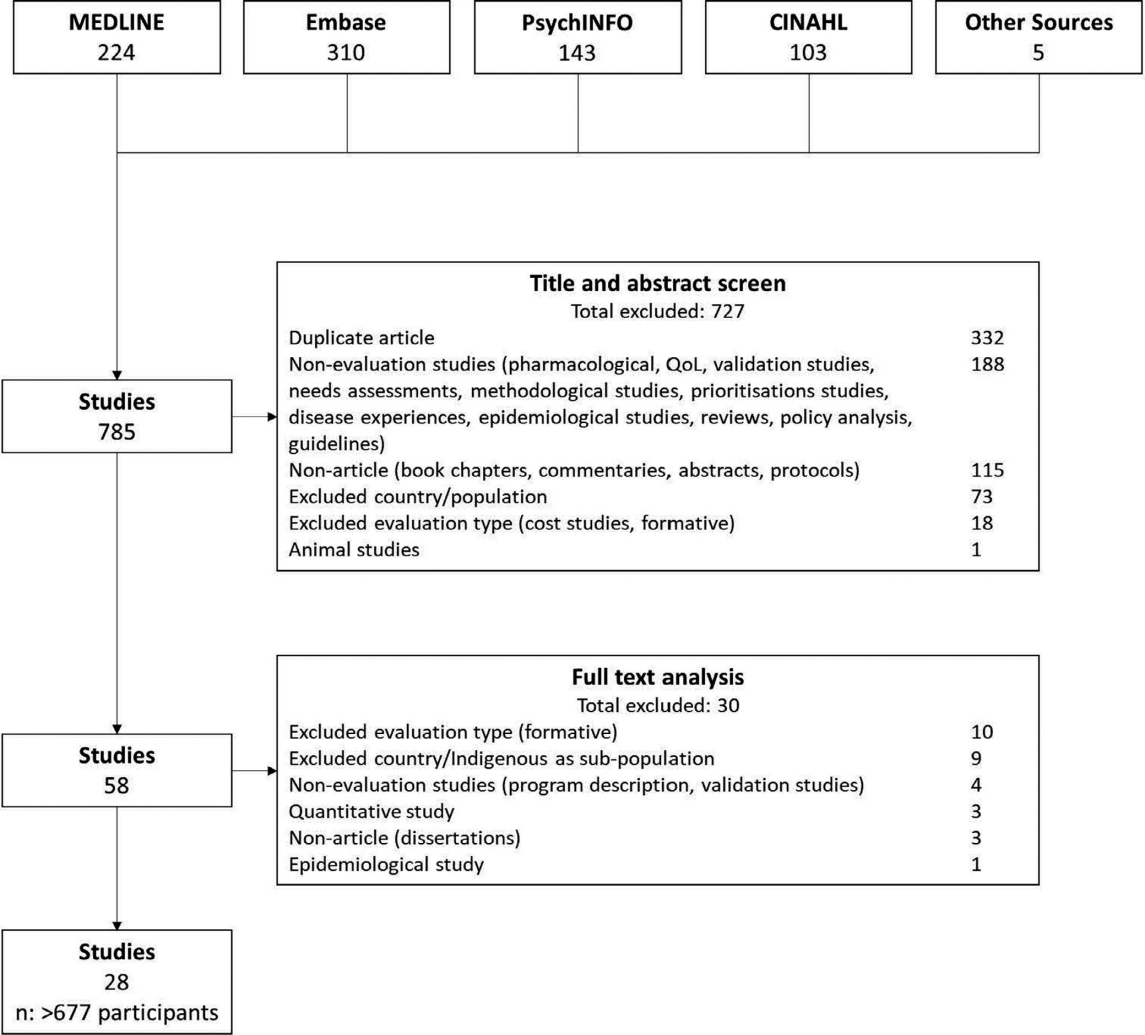
Search results

We included 28 studies involving more than 677 participants (seven studies did not report the number of participants) from Indigenous populations in four countries: Canada (36% studies), Australia (29%), United States (25%) and New Zealand (11%) (Figure 1). Stakeholders included programme participants, carers, staff, community members, community elders and representatives of community organizations familiar with the health programme. The type of evaluation studies from which the qualitative data were extracted was either outcome/impact evaluations (36%), process evaluations (29%) or mixed evaluations (36%). Mixed evaluations were mostly process and outcome/impact evaluations. The qualitative methods used in the studies included interviews, focus groups, open‐ended questionnaires and Yarning (an Indigenous method where the researcher develops an accountable relationship with the participant and they journey together visiting places and topics of interest relevant to the research study).^18^ Overall, the intervention programmes covered areas of mental health (including substance abuse and self‐harm), maternal health, chronic disease, lifestyle, infectious diseases and injury. The most common evaluations were of mental health programmes, 11 (39%) of the 28 studies. Additional characteristics of the included studies are provided in Table 1.

**TABLE 1:** Characteristics of included studies (N = 28)

Study ID	Year	N	Stakeholder group(s)^a^, ^b^	Age	Health focus	Data collection	Analysis
Australia							
Jan, S^19^	2004	35	Participants, community organizations, staff	Not stated	Maternal health	Focus groups, interviews, questionnaires	Thematic
Dimer, L^30^	2012	Not stated	Participants	Not stated	Cardiovascular disease	Interviews, yarning	Not stated
d'Abbs, P^7^	2013	32	Staff, community organizations	Not stated	Alcohol abuse	Interviews	Framework
Davey, M^31^	2014	51	Participants, staff	Not stated	Cardiopulmonary rehabilitation	Questionnaire	Thematic
McCalman, J^48^	2015	28	Participants, staff, family	Not stated	Maternal health	Focus groups, interviews	Thematic
Carey, T^23^	2016	20	Participants, family, community organizations	Not stated	Palliative care and chronic disease	Interviews	Interpretive phenomenological
Clapham, K 24	2018	34	Participants, staff, service providers	Not stated	Injury	Interviews	Framework and thematic
Skerret, D^49^	2018	49	Participants	Not stated	Suicide	Focus groups	Thematic
Canada							
Miles‐Tapping, C^50^	1994	10	Participants, staff	Not stated	Chronic obstructive pulmonary disease	Interviews	Not stated
Verrall, T^51^	2005	45	Participants, key informants	Not stated	Iron deficiency	Questionnaire	Not stated
Rosecrans, AM^28^	2008	34	Participants, staff, family, community	Not stated	Diabetes	Interviews	Not stated
Brussoni, M^25^	2012	42	Staff, community	Not stated	Injury surveillance	Focus groups, interviews	Thematic
Kiepek, N^52^	2015	91	Participants	Not stated	Opioid dependence	Interviews	Not stated
Rawana, J^53^	2015	15	Participants, programme developers	Not stated	Mental health	Interviews	Interpretive phenomenological
Pauly, B^20^	2016	11	Participants, staff	Not stated	Alcohol abuse	Interviews	Constant comparative
Nadin, S^54^	2018	29	Participants, family, staff	Not stated	Palliative care	Focus group, questionnaire	Thematic
Firestone, M^21^	2019	17	Participants, staff, community	Not stated	Well‐being and mental health	Focus groups, interviews	Thematic
Thomas, G^29^	2013	11	Participants	Not stated	Substance abuse	Interviews	Not stated
New Zealand							
Brewin, M^55^	2002	Not stated	Key informants	Not stated	Injury	Interviews	Not stated
Brewin, M^27^	2004	Not stated	Key informants	Not stated	Injury	Interviews	Not stated
Hamerton, H^26^	2012	Not stated	Participants, community	Not stated	Healthy lifestyle	Focus groups, interviews	Thematic
United States							
Bouey, P^56^	2000	18	Staff, participants, key informants	Not stated	HIV	Focus groups, interviews	Not stated
Nelson, K^57^	2011	Not stated	Participants	Not stated	HIV/AIDS, hepatitis, substance abuse	Interviews	Thematic
Goodkind, J^33^	2012	18	Participants	7‐17	Violence, trauma and alcohol abuse	Interviews	Thematic
Bosma, L^22^	2014	33	Participants, staff, community	Not stated	Commercial tobacco smoking	Interviews	Thematic
Rasmus, S^32^	2014	35	Participants, staff, community	Not stated	Suicide and substance abuse	Focus groups, interviews	Thematic
Chico‐Jarillo, T^58^	2016	11	Participants	Not stated	Healthy lifestyle	Focus groups	Thematic
Redwood, D^34^	2016	8	Staff	Not stated	Cancer	Interviews	Content

^a^ Group descriptions correspond to what is reported in the studies.

^b^ Participants are those who participated in the health programme.

### 3.2 Comprehensiveness of reporting

The comprehensiveness of reporting was highly variable with studies reporting 0 to 25 items on the COREQ checklist (Table 2). Eighteen (64%) studies reported on the interviewer or facilitator, 18 (64%) provided information on the methodology and/or theoretical framework used, 22 (79%) provided details on the sample size, 17 (61%) reported on the method of approach used for participant selection, and 20 (71%) studies included participant quotations.

**TABLE 2:** Comprehensiveness of reporting (N = 28)

Item	Studies reporting item	No. of studies (%)
Personal characteristics		
Interviewer/facilitator	7, 31, 32, 33, 48, 49, 52, 55, 56, 57	18 (64)
Credentials	19, 20, 21, 22, 23, 24, 52, 55, 57	13 (46)
Occupation	19, 20, 21, 22, 23, 24, 52, 55	12 (43)
Gender	20, 21, 22, 23, 24, 27, 31, 32, 55	9 (32)
Experience and training	19, 20, 21, 23, 24, 31, 32, 33	8 (29)
Relationship with participants		
Relationship established	22, 23, 24, 26, 29, 31, 32, 50, 55	9 (32)
Participant knowledge of the interviewer	21, 23, 24, 26, 29, 31, 32, 33, 50	9 (32)
Interviewer characteristics	19, 20, 21, 22, 23, 24, 55	10 (36)
Theoretical framework		
Methodological orientation and theory	19, 20, 21, 32, 33, 34, 48, 49, 51, 53, 54, 55	18 (64)
Participant selection		
Sampling	7, 48, 49, 50	15 (54)
Method of approach	7, 31, 32, 33, 48, 50, 52, 55, 56	17 (61)
Sample size	7, 19, 20, 21, 22, 23, 24, 25, 29, 31, 32, 33, 34, 48, 49, 50, 51, 54, 55, 56, 58	22 (79)
Non‐participation	7, 20, 22, 24, 33	5 (18)
Setting		
Setting of data collection	20, 21, 22, 23, 24, 25, 48, 50, 52	12 (43)
Presence of non‐participants	25, 32, 50	3 (11)
Description of sample	7, 20, 24, 31, 33, 34, 48	7 (25)
Data collection		
Interview guide	23, 24, 29, 32	4 (14)
Repeat interviews	21, 32	2 (7)
Audio/visual recording	19, 20, 21, 22, 23, 24, 48, 53, 54, 56	15 (54)
Field notes	25, 28, 50, 58	4 (14)
Duration	19, 20, 23, 24, 33, 48, 50, 54	8 (29)
Data saturation	48	1 (4)
Transcripts returned	22	1 (4)
Data analysis		
Number of data coders	20, 21, 22, 23, 48, 49	11 (39)
Description of the coding tree	25, 28, 32, 33, 34, 53	6 (21)
Derivation of themes	19, 20, 21, 22, 23, 24, 25, 48, 53	14 (50)
Software	20, 21, 22, 23, 24, 25, 48	11 (39)
Participant checking	21, 33	2 (7)
Reporting		
Quotations presented	19, 20, 21, 48, 50, 51, 52, 53, 54, 55, 57	20 (71)
Data and findings consistent	19, 20, 21, 48, 51, 53	15 (54)
Clarity of major themes	19, 20, 21, 22, 23, 24, 48, 51, 53, 55	16 (57)
Clarity of minor themes	19, 20, 21, 23, 32, 34, 48, 53	8 (29)

### 3.3 Synthesis

**FIGURE 2:**
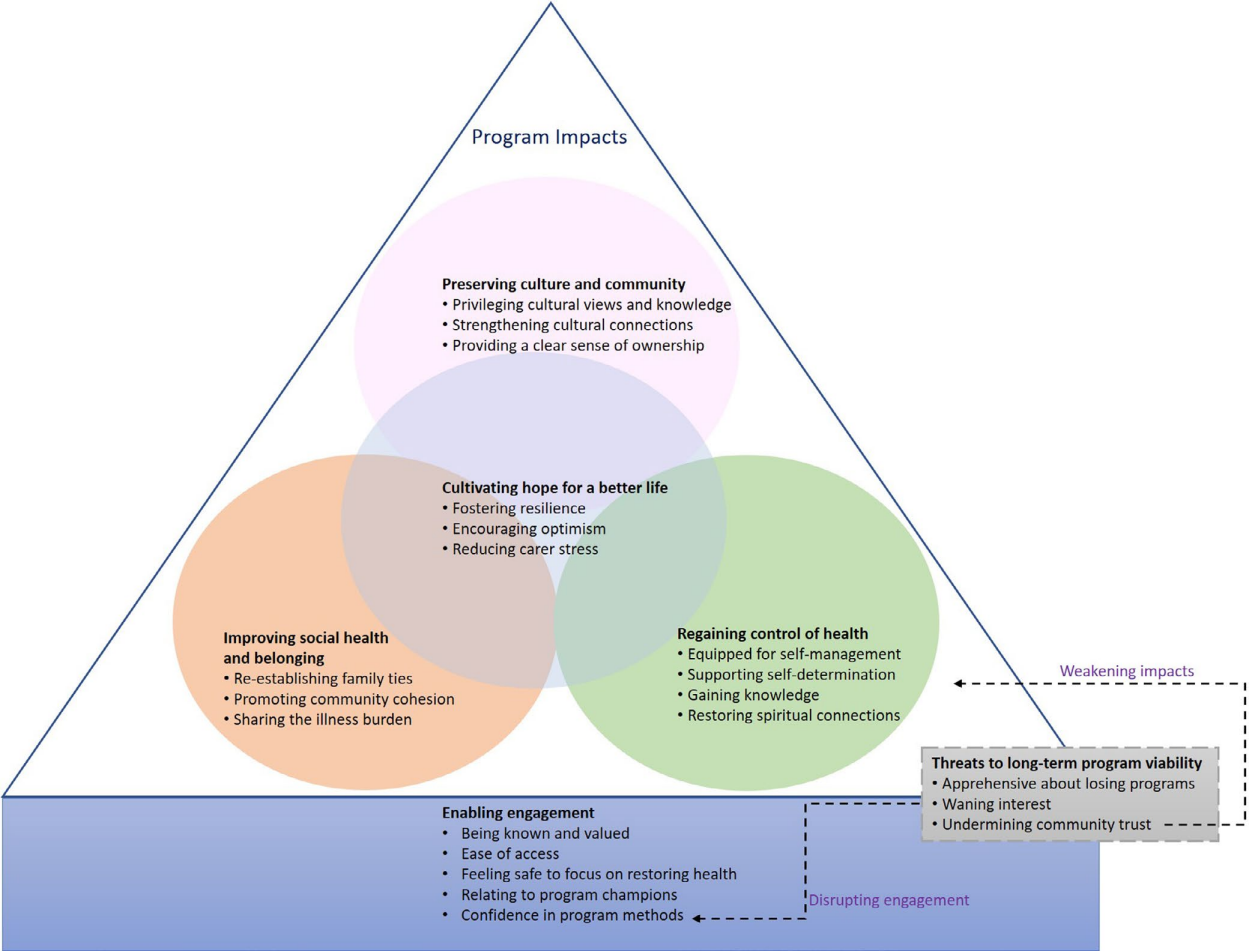
Thematic schema. Interventions that enabled engagement by providing ease of access to health services, ensuring participants felt known, valued, safe, confident in the programme's methods to address their health condition, and included programme champions were valued. Once engaged, participants regained control of their health, experienced improved social health and sense of belonging and felt that their participation contributed towards preserving their culture and community. Participants experienced these impacts throughout the life of the programme adding to a greater sense of hope around achieving health goals. When the long‐term programme viability was threatened, engagement was disrupted and confidence diminished in health programmes. The potential to achieve important individual and community outcomes was weakened as trust was eroded.

We identified six themes: enabling engagement, regaining control of health, improving social health and belonging, preserving community and culture, cultivating hope for a better life, and threats to long‐term programme viability. Each theme and sub‐theme is described below with selected quotations provided in Table S2. Concepts specific to a stakeholder group, type of intervention or setting are described accordingly. A schema to show the conceptual links between the themes is shown in Figure 2.

#### 3.3.1 Enabling engagement

Stakeholders observed that to facilitate engagement, participants required health interventions to identify and address barriers to accessing health services particularly those related to social and economic disadvantage.

##### Being known and valued

Given the history of mistreatment from health services experienced by many Indigenous peoples, participants felt that to build trust, staff should be welcoming, respectful and show genuine interest in their lives. One Australian Aboriginal participant of a midwifery service felt ‘at home’ and comfortable to ‘talk about any problems’ she had.^19^ Participants expected to bond with staff to be able to engage in the programme—‘I think it's a pretty good program. If it wasn't, I would've left a long time ago. Here they respect you.’(Canadian First Nations participant of a managed alcohol programme).^20^ Staff acknowledged the importance of a ‘client centred view’ and the need to provide services where participants felt respected and accepted as individuals.^20^, ^21^

##### Ease of access

Participants needed support to address economic^21^, ^22^, ^23^, ^24^ and environmental barriers—‘Transport makes it easier – if there wasn't no transport I wouldn't go for antenatal check‐ups,’ (Australian Aboriginal participant of a midwifery service).^19^ Participants, particularly those caring for children, needed support to attend health appointments because it was difficult to find someone to ‘rely on to look after your children.’^19^

##### Feeling safe to focus on restoring health

Feeling safe physically and culturally enabled participants to focus on restoring their health. Participants explained that cultural safety was about being comfortable to feel vulnerable—‘you don't have to explain yourself – don't have to feel defensive,’ (Australian Aboriginal participant of a midwifery service).^19^ Cultural safety for Canadian First Nations participants was described as fully understanding and appreciating the experiences of Indigenous communities and being able to use that knowledge to inform how services are delivered—‘I mean having that shared understanding and knowledge around the Indigenous population, the damages of residential schools and the impact of genocide… it's damaging to have to continuously tell your story over and over and over again. And entering a space where you don't have to do that and you can just participate is as safe as a space can get.’(Key informant for a Canadian First Nations community‐based wellness programme).^21^

##### Relating to programme champions

Participants were interested in programmes that involved respected community leaders who they could relate to and who inspired them to achieve their health goals without making them feel patronized.^22^, ^24^, ^25^ A Māori participant of a New Zealand nutrition programme learned from staff who she could relate to and who were approachable rather than judgemental—‘We know we are big … and we do know why, and so she [programme educator] comes in at a level where we want to engage in what she is doing… she is very humble, she is from our Hāpu [sub‐tribe].’^26^

##### Confidence in programme methods

Community members perceived that participants felt more confident in programmes that demonstrated tangible progress.^26^ One Māori community member observed that a community injury prevention programme had contributed to ‘a gradual shift in mind‐sets and attitudes towards not drinking and driving’^27^ among members of the community as they chose to adopt healthier behaviours that promoted health and well‐being.

#### 3.3.2 Regaining control of health

Participants wanted programmes which empowered them and helped them regain control over their health, as they defined it, and supported their approaches to achieving healthy lives.

##### Equipped for self‐management

Participants appreciated programmes that taught and provided resources to develop coping strategies and self‐management skills—‘I probably wouldn't have thought about half of the things that I got in the kit…some of the information, like, you just don't usually receive it unless you have some help there and some support,’ (Australian Aboriginal participant of a child‐safety programme).^24^ Participants also wanted to be confident about applying what they had learnt after leaving the programme. ‘They [Daruk] did give me confidence – I talk to them about something I'm worried about and they explain it in a different way and tell you how to go about it – and I feel alright then and I'll do it like that,’ (Australian Aboriginal participant of a midwifery service).^19^

##### Supporting self‐determination

In some communities, programmes helped to dismantle and challenge beliefs that kept people from exercising their individual power and capacity to improve health outcomes, by strengthening awareness over the aspects that they could control. ‘I really hadn't considered that we could be doing things to prevent injuries. I thought that accidents just happened,’ (Māori participant of an injury prevention programme).^27^ One staff member of a diabetes prevention programme for a Canadian First Nations community observed that participants wanted to feel supported in reframing their role in improving their well‐being—‘It's brought some information forward that the children hadn't been aware of before. And if nothing else, it's making them more aware that they need to be more physically active. Now they're talking about grandparents and other family members who are diagnosed already with diabetes, and some who have complications from it. They're putting it together that ‘whoa, I can do something about this.’^28^

##### Gaining knowledge

Providing information that was relevant and easy to understand meant that participants could gain the knowledge they sought to improve their health. ‘E [staff member] uses a certain language the women understand – they can relate to it – they seem to understand and accept things a lot better if it's in their terminology,’ (staff member at an Australian Aboriginal midwifery service).^19^

##### Restoring spiritual connections

Participants perceived the health and spiritual journey as intricately intertwined and considered both as necessary for health and well‐being.^20^, ^29^ A ‘key informant’ for a Canadian First Nations wellness programme observed that it was important for participants to view programmes as supportive of their spiritual journey and that it ‘validates their spirit.’^21^

#### 3.3.3 Improving social health and belonging

Participants felt some programmes helped them ameliorate the isolating effects of ill health and facilitate the process of restoring essential social connections that provide a sense of belonging.

##### Re‐establishing family ties

Family relationships could become strained as a result of ill health and participants appreciated programmes that facilitated reconnection with family. This reconnection helped support them in their health journey, ensuring they didn't feel isolated and removed from their social networks. ‘Well, it feels a lot better now… It wasn't like that when I was drinking. They look at me and they say oh he's drunk and let's go someplace else. Like when they come here they just give me hugs, take pictures, talk,’ (Canadian First Nations participant of an alcohol management programme).^20^

##### Promoting community cohesion

Some programmes became a ‘community meeting place’^30^ and stakeholders noted a ‘comradeship that develops between participants and staff…,’^31^ as they worked together to improve the health of the community. Participants felt that there were additional community benefits when health issues were addressed in partnership with the community. This is particularly true among communities where people felt disconnected and distant from each other and where generational gaps were evident—‘The Elders and young people meeting together is the best part of this programme. Young people, at the start, felt they were very far away. But we start telling about the right way to do things and it start opening things up– and it's bringing out the Elders in a positive way,’ (key informant for a Yup'ik Alaskan Native mental health programme).^32^

##### Sharing the illness burden

When the experience with illness was overwhelming and compounded by other daily struggles, participants valued spaces where they were around other people and staff who could relate to their experiences and the impact that an illness had on their lives. ‘Hearing others talk and share – that has really encouraged me to talk. The more we got involved the easier it became to speak in public… And to share my feelings and hear myself speak – it helped me to accept and go on and heal…,’ (Yup'ik Alaskan Native participant of a mental health programme).^32^

#### 3.3.4 Preserving community and culture

Programme activities that aligned with efforts to maintain community and culture reassured participants of the programme's commitment to community values.

##### Privileging cultural views and knowledge

Stakeholders felt that participants wanted health services to use approaches that validated their identity and contributed towards the community's collective efforts to decolonize how health was delivered and ‘do it our way.’^32^ Participants wanted programmes to implement health approaches that reinforced existing cultural traditions and knowledge which promote health and well‐being.^32^ One Maori participant of a nutrition programme highlighted the differences between what she perceived as a cultural Maori approach to an exercise programme versus the dominant culture approaches which were perceived as too rigid—‘You see, what funders need to take note of is the inclusive way in which we Māori exercise. It involves fun, laughter and the opportunity for us to all to laugh at ourselves.’^26^

##### Strengthening cultural connections

Participants perceived programmes which included learnings about culture and community as important for filling knowledge gaps around cultural identity. A Native American participant of a mental health programme perceived that the education about her culture had enriched her life by restoring her self‐confidence to confront health issues in a way she could not have done before. This was an experience she wanted to share with her child—‘I've learned the history of [community name]. I've learned how to cope with things in traditional ways because I want her [daughter] to have more knowledge of the traditional ways than I've never had. I want her to know where her roots are, who her family is, and I never got that opportunity.’^33^

##### Providing a clear sense of ownership

Participants actively took responsibility for programmes that demonstrated community involvement in decision‐making processes which shaped the programme's focus and direction and ensured that the programme's objectives were aligned with community priorities.^22^, ^27^ ‘It belongs to the community……It is our project, ours together,’ (community member of a Yup'ik Alaskan Native mental health programme).^32^

#### 3.3.5 Cultivating hope for a better life

Stakeholders observed how some programmes impacted participants' and carers' attitude towards a life affected by ill health.

##### Fostering resilience

Participants valued programmes that used strength‐based approaches, to help them redirect their focus from the difficult situations caused by ill health to their capacity to achieve a healthier life and overcome the challenges—‘I really faced myself,’.^29^ One participant of a Canadian First Nations mental health programme perceived that participation emboldened them to ‘speak up’ for themselves rather than ‘shrink away’ ^20^ from conflict.

##### Encouraging optimism

Participants appreciated programmes that helped them overcome feelings of futility and inspired them to develop an optimistic outlook on their life, giving them hope that they could have an improved quality of life and helping them to visualize it– ‘this program … has given me hope and has allowed me to really think what I wanna do with the rest of my life…there's a horizon waiting for me.,’ (Canadian First Nations participant of a mental health programme).^20^

##### Reducing carer stress

For programmes addressing chronic illness, participants felt that providing respite reduced carer burden and anxiety and enabled them to cope with care needs. ‘No‐one would be at home, my wife would be working … she worry, that's why she wanted to find a place where I can stay for the day…,’ (Aboriginal Australian participant of a respite facility).^23^

#### 3.3.6 Threats to long‐term programme viability

Due to bad experiences with programmes, stakeholders had concerns about sustainability.

##### Apprehensive about losing programmes

Experience with programmes being cut prematurely left stakeholders feeling fearful that community members may miss out and that the cycle of disadvantage would continue. ‘I've seen this many, many times in the Indian Health Service and now in the Alaska Native Health System is that when the grant for a program starts to shrink, the program also begins to shrink. And when the grant goes away, the program goes away and it has nothing to do with how important that program is to the health of the population or how successful the program is,’ (key informant for an Alaskan Native cancer outreach programme).^34^

##### Waning interest

Stakeholders perceived that in some communities, the initial interest in a health programme could be attributed to the novelty of the initiative, ‘At the beginning, it was good because the program was new…’ (community member of a Yup'ik Alaskan Native mental health programme).^32^ However, often, participation decreased when the novelty wore off.

##### Undermining community trust

Stakeholders felt disappointed by short‐term programmes and perceived that they eroded community trust in the health system. ‘It's not a one‐time effort, it has to be done over and over,’ (stakeholder from a Native American tobacco cessation programme).^22^

## 4 DISCUSSION

Indigenous participants experienced a complex interplay between factors affecting their engagement in health programmes and the outcomes of participation. They perceived that access to health programmes was facilitated when they felt known, valued, safe and confident in the methods used to address their health condition, and when interventions employed ‘champions’ they could relate to. Once engaged, participants were able to regain control of their health. They felt that their participation contributed towards preserving culture and community leading to improved social health and a greater sense of belonging. When these outcomes were successfully achieved throughout the programme, this added to a greater sense of hope about their lives. When the long‐term viability of programmes was threatened, engagement was disrupted as confidence diminished and the potential to achieve the individual‐ and community‐level outcomes were weakened with the loss of trust.

On an individual level, the key message from all Indigenous participants in this review was how Indigenous people from the United States, Canada, Australia and New Zealand feel while attending health services matters and contributes to their social, emotional and spiritual well‐being. These feelings should be acknowledged, respected and responded to appropriately because they add to the participant's overall perceptions of health, reflecting the holistic views of health held by Indigenous people often in contrast to non‐Indigenous populations in the same countries.^35^ Participants from all four nations reported on how the constraints of their socio‐economic status affected their access to transport and childcare services so they could access health services. Community‐level outcomes such as community cohesion were also reported in all four countries. These were often an unintentional result and showed how some health programmes produced value for the whole community. Other community‐level outcomes related to preserving community and culture.

Similarities in the colonial histories of the United States, Australia, Canada and New Zealand have led to shared and well known experiences of discrimination, mistreatment and disenfranchisement.^36^ This may explain the consistency with which the experiential outcomes around social, emotional and spiritual well‐being and the community‐level outcomes were reported across Indigenous communities and countries. Moreover in these nations, Western epistemologies in approaches to health service delivery dominate and inform processes vulnerable to practices, which if left unchecked, deny Indigenous peoples their humanity and de‐legitimizes their ways of knowing.^37^

While the experiences of mistreatment are not identical, nor the effects of the mistreatment the same for individuals or communities, the ‘disempowered positions’^38^ of Indigenous communities in the four countries have resulted in profound and strikingly similar needs across health‐care programmes and systems. Addressing the health‐care needs of Indigenous people requires a sustained commitment by governments to eliminate systematic institutional discrimination. National health policies should promote programmes that have clear processes to identify and address mistreatment identified by the Indigenous communities.

In this review, participants pointed to the mechanisms and processes within health programmes that facilitated achieving positive experiential outcomes. Broadly, participants from all four countries often attributed experiential outcomes relating to social, emotional and spiritual well‐being to their interactions with staff and actions taken by staff. This is consistent with previous research which showed that positive interactions with staff promote engagement, while poor treatment has resulted in Indigenous people avoiding health care.^39^ Participants in our review also reported on how staff influenced the physical environment of health care by managing spaces in a way that allowed participants to feel comfortable and safe. The availability of staff who possess the requisite interpersonal skills and cultural respect is key to ensuring Indigenous participants achieve the valued social and emotional outcomes. However, access to facilities with skilled staff varies according to where Indigenous people are able to access health‐care services.

In the United States, the Indian Health Service (IHS) supports health services on the Reservations of Federally recognized Native American communities which may be close to urban areas or in rural locations. In contrast, Native Americans who do not live on reservations use mainstream health services options.^40^ Similarly in Australia, Aboriginal Community Controlled Health Organisations (ACCHOS) offer health services to Aboriginal and Torres Strait Islander communities mostly in rural and urban communities, with limited access for remote communities.^40^ In Canada, urban‐based First Nations people do not generally access Indigenous specific services, and in New Zealand, many Maori can only obtain some health services from mainstream services.^40^ This diversity of health access for Indigenous communities makes Indigenous people vulnerable to encounters with staff who do not have the requisite skills to meet their social, emotional and spiritual needs.

Strategies to improve the availability of qualified staff working in health services for Indigenous communities have been implemented in all four countries. The main focus has been on increasing Indigenous health staff through training and education. The IHS grants scholarships to Native Americans students pursuing health careers and in Australia government funded programmes like the Remote Area Health Corps support health workforce capacity building.^40^, ^41^ Despite these programmes, shortages of skilled labour with relevant clinical skills persist and recruitment criteria around interpersonal skills remains peripheral.^42^ Government policies should support capacity building initiatives that go beyond generic ‘cultural sensitivity training’ to those that develop the interpersonal skills identified by communities as important.^43^ Health services should conduct reviews of internal processes to identify the social and emotional needs of programme participants to produce data that informs training needed to operate in their community.^44^ Further research should investigate the effectiveness of health education curriculums in providing care to Indigenous people.

Consistent with other research, our review shows that addressing costs borne by individuals can also improve access to, and confidence with health programmes. Both the costs and safety concerns of public transport impede access to health services for Indigenous people.^45^ The burden of meeting financial needs as they relate to health‐care access often rests with local health service providers in all four nations. Although national schemes to assist with such costs are available in the United States, Canada, Australia and New Zealand, often trade‐offs have to be made due to budget limitations.^40^

In the United States, efforts to reduce the economic burden of care for Native Americans are constrained by an underfunded IHS which remains the main source of funding for health expenses not covered under national schemes.^46^ Until Federal funding for the IHS matches the need for health services and the resources required to reduce barriers to accessing health care, disparities in health outcomes experienced by Native Americans will persist.^46^ Cost also presents a barrier to accessing care in Canada and New Zealand, with the cost of transport being a major barrier for rural and remote communities.^40^ In Australia, Federal funding support through the Indigenous Australians' Health Programme aims to assist with the cost of receiving care.^41^ However like the United States, available resources do not match the health‐care needs and demand for services. Allocating resources should be based on an understanding of the varying needs of communities and participants to ensure targeted use of finite resources that are relevant to individual communities and to avoid negatively impacting engagement.

The negative effects of short‐term programmes on community affected communities from all four countries in our review. Programmes that ended prematurely reinforced a narrative of distrust and disenchantment towards health services. The uncertainty that came with new programmes introduced into some Indigenous communities caused frustration. Policies that support stable funding for Indigenous health programmes are needed.

Our review supports the use of qualitative methods within process evaluations, particularly in identifying and understanding the value of the medium and long‐term outcomes of health programmes.^47^ Our assessment of the comprehensiveness of reporting from the evaluation studies in our review revealed poor reporting of the qualitative data collection and analysis processes which supports the need to promote the use of frameworks to improve the quality of reporting of qualitative evaluations of Indigenous health interventions and programmes.^5^, ^47^

The results from this review reveal the depth and diversity of stakeholder perspectives on participants' experiences with the implementation of health programmes and subsequent impact across a range of settings. We identified how the contextual factors experienced by Indigenous communities affect engagement in complex interventions among communities from Australia, Canada, the United States and New Zealand. We also outline the reasons for the outcomes reported by stakeholders from these interventions and developed a new analytical framework that captures the diversity and depth of data on the factors affecting engagement and the experience of programme impacts across different populations and health‐care contexts.

However, there were some potential limitations. We excluded non‐English articles due to resource limitations. The review combined studies from different Indigenous communities with different cultural heritages, socio‐economic and political experiences and only included evaluations that were published in peer‐reviewed journals which may not reflect the experiences of participants with all existing health programmes. This review establishes a foundational basis on which additional research can build. Where sufficient data exist, further research that is localized could be conducted to provide specific reasons for outcomes and their value to that community and examining evaluations from the grey literature may expand knowledge around participant experiences.

## 5 CONCLUSION AND IMPLICATIONS

These findings describe how approaches to health‐care programming contribute towards achieving health outcomes for Indigenous participants and importantly community‐level benefits. The prominence of social, emotional and spiritual well‐being as important aspects of the health journey for participants in this review highlights the need to reframe evaluations of health programmes implemented in Indigenous communities away from assessments that focus on commonly used biomedical measures. The ability to define the specific aspects of health services that impact engagement, such as staff possessing the interpersonal skills to empower communities to achieve their health goals on their own terms, emphasizes the value of participant‐reported outcomes in evaluations. Evaluators, in consultation with the community, should consistently assess the ability of health professionals to meet community needs and expectations throughout the life of the programme. Evaluations that include qualitative data obtained from participants can provide decision‐makers with an important assessment of the impact of programmes both on participants and their communities.

## CONFLICT OF INTEREST

None to declare.

## AUTHOR CONTRIBUTIONS

SC conducted the search, screened and analysed the data and drafted the manuscript. JD independently reviewed and assessed included articles. AT, MH, MD, JC, SE and KH provided significant input into the conceptualization of the study, study design and analysis of the data. All authors critically revised and approved the final manuscript.

## Supporting information

Tab S1‐S2

## Data Availability

The data supporting the findings of this study are available within the article and its supplementary materials.
