# Supplementary material for: Stakeholder perspectives on the implementation and impact of Indigenous health interventions: A systematic review of qualitative studies
Source: Health Expect. 2021 Mar 17;24(3):731–43. doi: 10.1111/hex.13230 (PMC8235882; doi:10.1111/hex.13230)
Supplement: Supplementary file 1 — Tab S1‐S2 [file HEX-24-731-s001.docx]

**SUPPLEMENTARY**

**Supplemental Table 1. Search strategy**

| **Database^a^ (Ovid)** | **Search Terms^b^** |
| --- | --- |
| MEDLINE, PreMEDLINE, Embase, PsycINFO | (1) Aborigin*or Indigenous or (First adj nation*) or (First adj People*) or Oceanic Ancestry Group/ or (Torres adj Strait adj Islander*) or Maori* or Arawak* or Amerindian* or Tutong* or Belait* or Dusun* or Murut* or Kedayan* or Bisaya* or ("Iban adj people*" or "Iban adj population*" or "Iban adj community*" or Ibans) or Penan* or Inuit* or Metis or Inuvialuit* or Nunangat* or Inupiat* or Mapuche* or Aymaras or Quechuas or Rapanui or Yamana or Alacaluf* or (Sami or Samis) or Saami* or Lapps or Laplander* or (Greenlandic adj Inuit*) or Chamorro* or Chamoru* or Ainu* or (Okinawan or Okinawans) or Ryukyuan* or (Kanak or Kanaks) or ((Taino adj Indian*) or Taino*) or (Indigenous adj Malay*) or (Plains adj Aborigin*) or (Taiwanese adj Aborigin*) or (Austronesian adj Taiwanese) or Guarani* or (Native adj American*) or (American adj Native*) or (American adj Indian*) or (Indian adj American*) or (Native adj Alaska*) or (Alaska* adj Native*) or Eskimo* or yupik* or (Aleut or Aleuts) or Inuk* or Cherokee* or Navajo* or Choctaw* or Sioux* or Chippewa* or (Apache adj people*) or Apaches or Blackfeet or Iroquois or (Iroquois adj Nation) or Pueblo* or american native continental ancestry group/ or alaska natives/ or indians, central american/ or indians, north american/ or indians, south american/ or inuits/ or (Native adj Hawaiian*) or (Hawaiian adj Native*) or (native adj people*) or (native adj population*) |
|  | (2) (cost adj analys#s) or (cost adj consequence) or (cost adj effectiveness) or (cost adj utilit*) or (cost adj benefit*) or (cost adj minimi#ation) or (cost adj outcome*) or (quality adj2 life) or (health adj2 quality adj2 life) or (quality adj adjusted adj life) or QoL or QALY* or QUALY* or (Disability adj adjusted adj life) or DALY* or (process adj utilit*) or (health adj evaluation*) or (project adj evaluation*) or (program adj evaluation) or (programme adj evaluation) or (health adj service adj evaluation) or (health adj promotion adj evaluation*) or (economic adj evaluation*) or (process adj evaluation*) or (implementation adj evaluation*) or (impact adj evaluation*) or (outcome adj evaluation*) or (outcomes adj evaluation) or HRQol or (life-year adj gained) or (life-years adj gained) or (life-year adj lost) or (life-years adj lost) or (life-year adj saved) or (life-years adj saved)  (3) (qualitative) or (interview) or (focus group) |
| ^a^ Searches were modified for CINAHL; ^b^ All terms were searched using text words (tw) and combined to extract the final search; (1) Search terms used to identify Indigenous populations; (2) Search terms used to identify health evaluation studies; (3) Search terms used to identify studies with qualitative data | |

**Supplemental Table 2. Selected illustrative quotations by theme**

| Theme | Quotes | Contributing Studies |
| --- | --- | --- |
| **Enabling engagement** |  |  |
| Being known and valued | “When I’m sitting at home, sometimes my family they go into town, they do shopping and they leave me alone, I mean, I’m on my own, and since I came here these other people from other town camps, I know we don’t talk much, but we get together and just sit around and watch TV… These people that work here they getting to know us so we like to make sometime tell story to one another, or sometime make joke of one another, so I’m happy yeah.” (participant)^1^ | ^1-7^ |
|  | “Daruk knows your background – they know your needs.” (participants)^6^ |  |
|  | “We will help you whatever shape you are in.” “So that holistic very client-centred view, very harm reduction-based focus which allows people to be where they’re at in whatever space they’re in and be part of the program and be respected in the program. People have let us know how important that is to them." (staff)^7^ |  |
|  | “What it meant to me is like they don’t trust me, like over here [MAP] they got a open mind and ‘be safe and come back’, ‘come back in one piece’…What I think is the workers there [residential treatment] they think right away “oh he’s gonna relapse, oh he’s gonna go do something stupid”. So I think that’s why they ask you those questions right off the bat, but here (MAP) they don’t ah ask any questions when you go somewhere. It’s like “I gotta go meet some body; I gotta go do this”“ok bye, come back in one piece, be safe”.It’s almost like they’re giving all their trust in you, the workers here, it’s like they trust you and when I was at the [residential program] one day they did that to me. First thing I thought was after the twenty questions, walk out the door, get on the street, “they don’t trust me, I should just go drink” like ah the [residential program] they’re expecting you to fail. But here they got confidence in you.” (participant)^5^ |  |
| Ease of access | “I think because we’re putting food in their bellies, that in itself just breaks down so many resistances and people start laughing. When they’re around food they get a little bit dance-y and so I think that in itself just humanizes the experience of people coming together and that’s what we’re trying to do right across the province now is feed the people. Bring them in to feed them. That gets people through the doors and eliminates that clinical atmosphere. It just makes everything a lot more familiar and communal and comfortable. And I think that that’s huge for Four Winds [Niiwin Wendaanimak program].” (Key Informant)^7^ | ^1-3, 6-11^ |
|  | “The Aboriginal Medical Centre took me there, picked me up from my home, made sure I was there on time, waited for me, bought me back home. It was a lot easier.” (participant)^9^ |  |
|  | “I rang one time to say I couldn’t come to antenatal check-up because I didn’t get off work till 5 p.m. – so they waited till I could get there.” (participant)^6^ |  |
| Feeling safe to focus on restoring health | “Now I can talk about what happened to me without breaking down, without having to cry. Why? Because I went and got help for that. I had to go to my own culture to do that.” (Key Informant)^7^ | ^1, 5-7^ |
|  | “So but I heard it put really really nicely in one of the teachings and the Elder said, you know, this is a reminder for us to be humble, to recognize these warriors that are substance users because they’re doing that for us so we don’t have to. Just a reminder of what they’re giving up for the rest of us. And I thought that was so beautiful because that’s so true. It’s right in your face every day of where we could all be. And so it’s a gentle reminder to be humble and accept the gifts that you have and share them as well to help our struggling people.” (Key Informant)^7^ |  |
|  | "you feel safe, you feel like you’ve got a warm place to stay, and you know, some home.” (participant)^5^ |  |
|  | “Yeah, when I first moved in here, it was like moving into a prison, eh, but a month later I felt a lot safer. And the staff is there all the time. That’s what I like about this place. Yeah, there’s a lot of things I like about this place.” (participant)^5^ |  |
|  | “There was a few times that, when a resident was picking on me … and then I had to ask one of the staff to come and sit with me … and then I would tell him I wanted to go to sleep, and he’d stay here until she goes to sleep. So he would stay [with me] until she fell asleep. She doesn’t bother me no more.” (participant)^5^ |  |
| Relating to champions | “seeing community members who have been involved with this program leading the way with their exercising and healthy attitudes” (participant)^12^ | ^2, 6, 9, 12-15^ |
|  | “There is no more cream or [full fat] milk, cakes. We eat healthy all the time now. I have to lead by example and that was hard at ﬁrst, but having only healthy food has been beneﬁcial for not only me but my staff.” (community leader)^14^ |  |
|  | “Yeah, I saw some of the health changes when we had food at the health agency prepared by the women. They got their vegetables out of their garden; they had planted their own vegetables as well. There was a really neat older woman on the programme as well and she brought some really good skills with her about the old time cooking.” (community member)^14^ |  |
|  | “exciting to see people of your background doing positive things.” (participant)^15^ |  |
| Confidence in program methods | “I was completely comfortable when you know that there’s a qualified nurse there and friendly [staff member] making scones and pikelets, they make us feel really comfortable.” (Carer)^1^ | ^5, 12, 14, 16^ |
|  | “It’s making me [not] drink that stuff anymore, and hairspray. Because it- my liver is going, but when I have the wine here, I don’t wake up sick.” (participant)^5^ |  |
| **Regaining control of health** | |  |
| Equipped for self-management | “The program is… teaching us to be in a home. You know, not like what we’re used to, out on the street. Like re-learning how to be in a house with responsibilities: got to make your bed, do your laundry, sweep, wash the floor, do dishes, and of course, we’re starting to cook. Most of us I think are just re-learning domestic things that you would normally do in a home. It’s another one of the benefits that we get living here.” (participant)^5^ | ^1, 5, 8, 12, 14, 17-18^ |
|  | “That you can always go to someone and ask for help . . . problem solving to talk to someone older instead of handling it yourself.” (participant) ^17^ |  |
|  | “Talk about it and go outside . . . or talk to yourself about it . . . to always talk to someone if you’re mad or go walk around and calm yourself down.” (participant)^17^ |  |
|  | “Keep busy. Keep self away from friends. Right now, trying to keep myself healthy and clean and keep my life together. I’m with people who support me. I don’t lie or hide the truth. Focus on future goals.” (participant)^18^ |  |
| Supporting self-determination | “It’s brought some information forward that the children hadn’t been aware of before. And if nothing else, it’s making them more aware that they need to be more physically active. Now they’re talking about grandparents and other family members who are diagnosed already with diabetes, and some who have complications from it. They’re putting it together that ‘whoa, I can do something about this.” (staff)^11^ | ^5, 8-9, 11-12, 14, 17, 19^ |
|  | “We’ve got some high risk families who actually really don’t know the correct car seat and they don’t know about blind chords and they haven’t even thought about the toddler pool and the fact that their baby might drown in it, they actually haven’t even thought about it.” (staff)^9^ |  |
|  | “Sharing my knowledge with whānau, like the children. Get my whānau to buy into the project. Roll on effect like decreasing smoking. Through my knowledge my daughter has taken up a more healthy lifestyle riding her bike and walking. Generally the whānau are eating healthier.” (participant)^14^ |  |
|  | “Before I came here I wouldn’t care what I was wearing or what I ate….I used to crawl into dumpsters, get something to eat ah… start drinking anything.”. (participant)^5^ |  |
| Gaining knowledge | “Mom, McDonald’s, its junk food, I know it is not good for me.” (participant)^11^ | ^6, 8, 10, 13, 19-20,11, 21-22^ |
|  | “They’re very visual people, and this gives them something to see. You know the actual seeing the food being cooked, or having it right there so they can taste it and then see it. Whereas a lot of the pamphlets we have, it’s a lot of reading [.] But I ﬁnd that the food sampling gives them the opportunity to feel comfortable, a little more relaxed, and they seem to think of questions to ask as they’re eating.” (community member)^11^ |  |
|  | “iron-fortiﬁed cookies and cereal. I wouldn’t have known there was iron in these foods if I didn’t see these displays at the store.” (participant)^22^ |  |
|  | “Daruk tells you what you want to know.” (participant)^6^ |  |
|  | “I guess the way they explained it to make it more simple to them instead of using big words.” (participant)^21^ |  |
| Restoring spiritual connections | “An aspect of my work that I found the most challenging and the most unique, and I still think are what sets this apart from my other program in the City of Toronto for people who are Aboriginal is you’re combining harm reduction and you’re combining spirituality and First Nation teachings and bringing them together and sort of breaking down those barriers which disallowed people who were in the throes of using to participate in their own Native culture.” (Key Informant)^7^ | ^5, 7, 16^ |
|  | “I think the importance is that it validates their spirit. Our most marginalized people have been ostracized and booted out or paid off to get out of our communities. You know, so they’re used to rejection. So when it comes to our spiritual supports if we reject them there too, then that puts their spirit even lower than it already is. You know, so I think it’s important to validate their spirit but also to validate their right to the medicines their right to what makes them strong. Like if you deny that to them right off the hop, then why are you there to begin with?” (Key Informant)^7^ |  |
|  | “I was sick most of the time. Not only alcohol sick but like body sick, spiritually sick. I believe in my culture and my traditions and plus the creator and I lost that you know. I lost that part there where we would you know smudge in the morning and you know and say thank you to our creator and then somehow I just quit doing that. Quit praising, quit praising our creator, I used to be able to, you know, join the celebration, you know there’s pow wows and all that. I don’t even do that anymore you know, put on my regalia and go celebrate. But now I, I haven’t picked it up yet again but it’s like, like I’m slowly getting there. I don’t think you’re ever a whole person because there’s always something new that’s gonna make whole, you know fuller as a person.” (participant)^5^ |  |
|  | “I got my spirit back, for one. Nature, like it’s saying “wake up and smell the coffee.” Like it’s so beautiful outside, and where was all that all this time? You know, I was just living [with] a black cloud over me. And the black cloud’s been removed basically. Because life is a lot nicer than it ever was. You know? I go spirit bathing every morning.” (participant)^16^ |  |
|  | “I had no sense of spirituality before really, coming clean and sober even while I was going through, like AA and NA. They tell you to reach your higher power or whatever. I thought that was a bunch of bull. But after the retreats I’ve really opened up to spirituality big time. I smudge every night before bed. I pray. I, you know, I say thanks to whatever is out there, you know?” (participant)^16^ |  |
| **Improving social health and belonging** | |  |
| Re-establishing family ties | “My mom and my dad pay more attention to me now and they know that they have to respect one another and be kind to each one including me.” (participant)^17^ | ^5, 16-17^ |
|  | “I’ve learned that we have to work together. We have to communicate and communication is the main thing. We have a lot of difﬁculty in that because we think that we know what the other is talking about but we really don’t and then we do the wrong thing. Here [in the intervention] we communicate and I’m trying to do that and then to work at that because that’s the main thing for us.” (participant) ^17^ |  |
|  | “The communication is a lot better with my family, and we’re all pretty much getting…more involved with each other now than we used to be before. It [the program] really brought us all together . . .” (participant)^17^ |  |
|  | “I see how responsible adults they’ve grown up to be and you know, I missed all that, you know. And I feel sad about it, … at the same time I feel encouraged, like, they never gave up on me.” (participant)^5^ |  |
|  | “It’s opened up where I felt I had a door closed to allow and to be, to allow my close family members inside me. It’s a . . . I can’t describe it right now but, I see the changes in my grandkids’ response towards me, and they are always like, want to be around, around me and my wife so, it’s lots to tell you there. Safety I guess, from our change.” (participant)^16^ |  |
| Promoting community cohesion | “I’ve lived here over 30 years, I came from downriver, and I’ve lived here for a long time now, and it’s the hardest place I’ve lived. Other places, people share. The whole village shares. Everyone was like brother and sister or uncle and aunt. Then we moved here and it was like closed-in. And I want to say … This project seems to open up a place that once was closed. Now when I walk down the street people will say happy to meet you and smile. Children will say hi to Elders now. That opening and friendliness is different. There is more understanding now – about survival, nature and who we are and how we have to live.” (CPG member, local leader)^23^ | ^10-15, 17, 23^ |
|  | “The Elders and young people meeting together is the best part of this program. Young people, at the start, felt they were very far away. But we start telling about the right way to do things and it start opening things up– and it’s bringing out the Elders in a positive way. We saw a vision. We want to bring people together. And young people talk about the meetings and ask – when are we going again. It’s like a net we have neglected – if it needs mending to ﬁx the holes. This project is like a big net – we are catching people here in the community with this net – dead or alive and if dead they might come alive again – Ellangneq. It’s time to wake up. It’s time to come alive.” (CPG member, local leader)^23^ |  |
|  | “creates a positive image of [Aboriginals] among non-Aboriginals.” (participant)^15^ |  |
| Sharing the illness burden | “When someone gets really sick and has to go to the hospital, or someone is missing, or, whatever, there’s, like, a collective sense of, you know, worry, right? The whole house, kind of, goes into, you know, worry, right? Or concern, or grief, or whatever. So, yeah. ‘Cause they are all very connected as well. Small community, in, in a way, you know?” (participant)^5^ | ^1, 5, 12, 17, 23-24^ |
|  | “Hearing others talk and share – that has really encouraged me to talk. The more we got involved the easier it became to speak in public. And to share my feelings and hear myself speak – it helped me to accept and go on and heal. …Through this program I learn, I share and I put it into practice.’’ (CPG member, parent)^23^ |  |
|  | “Can I just say [staff member] and [staff member] are my friends.” (participant)^1^ |  |
|  | “When I’m sitting at home, sometimes my family they go into town, they do shopping and they leave me alone, I mean, I’m on my own, and since I came here these other people from other town camps, I know we don’t talk much, but we get together and just sit around and watch TV… These people that work here they getting to know us so we like to make sometime tell story to one another, or sometime make joke of one another, so I’m happy yeah.” (Participant)^1^ |  |
|  | “Yeah, we think of each other as a family. When there’s a new person that comes in we welcome them with arms open. And we see they need to be [guided] for the first couple of weeks and we take them and we teach ‘em. And we, ah, show them around and if they need something I’ll show them where to get it, where to ask for it.” (participant)^5^ |  |
| **Preserving community and culture** | |  |
| Privileging cultural views and knowledge | “Jerry [Mohatt] never try and tell us we can’t do this or we can’t do that. We do it our own way and they supported that.” (Elder)^23^ | ^7-8, 14-15, 23^ |
|  | “I always thought that Nanny had some wonderful ideas about looking after us but somehow these got lost in Mum’s generation. It’s neat that we are looking at these practices again now. I think it is what we need to help our whanau [extended family].”(participant)^8^ |  |
|  | “The teaching of non-interference is a world-view that allows others to experience and learn life lessons in their own way and in their own time. This way of being is complemented by harm reduction. Earth-based cultures always used a campfire. If a child is walking toward a campfire, we do not snatch them away and slap their hand. Instead, we walk closely behind allowing the child to reach out to the fire. The child will pull the hand back when they learn that fire hurts. They learn to respect fire. This is non-interference. The rocks around the fire pit may also serve as a physical barrier, thus harm reduction. Service-wise, this means that we take a strength-based approach that is “client-centered”, as opposed to having an agent of a foreign system diagnosing and prescribing or enforcing changes that make the prescriber feel better. As with everything in life, there are always limits. “We do the circle and it’s for just Native people. And what do we do there? We honour the circle, our culture, and it gives a person a chance to share, to pray or sing.” (Key Informant)^7^ |  |
|  | “She told me that she was taking the girls out to get kina (a kind of seafood). She would organise for when they would go, organise correct footwear, clothing and then she spoke about how good the kina was for them. For some, they didn’t even know what a kina was let alone the goodness from them, especially for iron. So it was an introduction to the local resources and the health beneﬁts.” (participant)^14^ |  |
|  | “Walking around the gym I guess for us would be a band-aid solution. I want to do two things: get people out walking and get rid of the dogs. So the only way to get rid of the dogs is to get something going like a walking group, and this way the chief and council can do action.” (staff)^11^ |  |
| Strengthening cultural connections | “There’s a deeper connection in the physical place itself. I think it goes down to that ancestral-level memory of our people and I think there’s different historical aspects of why this area and why these people are suffering in this area. So, it may have been the catastrophic traumatic experience of our people way, way back and you know, they’re pulling these people here for whatever reason.” (participant)^7^ | ^7, 17^ |
|  | “Also, historically this corner…there’s old photos I saw a couple of years ago from over a hundred years ago where this used to be an old bank. And there were Aboriginal members sitting on the front steps of this corner of the bank. So, there’s part of it (that is) land ownership. And so I think that that’s really important for people and you hear that all the time. We’ll have new staff members come in and people take a lot of pride in the fact that they have been here for years and years and they continue to come and this is their sort of space and they take great ownership over that. So yeah, so I think that that, you know, that’s a huge part of why people come.” (participant)^7^ |  |
|  | “I was interested in that [access to traditional teachings and language] just because I keep hearing… not just learning about yourself but also learning about other cultures and other people. It makes it a little easier to… I don’t know, interact or reach out or make new friends.” (participant)^7^ |  |
|  | “I’ve learned a lot about the [tribal] culture. Things that I really didn’t know. Also, I have more respect for my people.” (participant)^17^ |  |
| Providing a clear sense of ownership | “This is their clinic.” (stakeholder)^2^ | ^2, 8, 13-14, 23^ |
|  | “I’m pleased we have our own scheme operating, it’s good to have another provider other than Plunket. Some of our whanau never get involved with Plunket.” (participant)^8^ |  |
|  | “.. . Sharing my knowledge with whānau, like the children. Get my whānau to buy into the project. Roll on effect like decreasing smoking. Through my knowledge my daughter has taken up a more healthy lifestyle riding her bike and walking. Generally the whānau are eating healthier.” (participant)^14^ |  |
| **Cultivating hope for a better life** | |  |
| Fostering resilience | “This program and any other program that educates, empowers Aboriginal people to gain beneficial results regarding their health is nothing but a positive for community and families. Let there be more.” (staff)^12^ | ^1, 5, 8, 12, 16-19^ |
|  | “I’m learning how to speak up for myself other than just shrink away when, you know, somebody doesn’t… agree with what I’m doing.” (participant)^5^ |  |
|  | “I’m more into my work, and I’m also more into my daughter, who this is for. You know, we’re doing okay as far as getting along, and her self-esteem is a lot better. This program has really helped her with her self-esteem.” (participant)^17^ |  |
|  | “[The retreat] affected my life in giving me another chance at life rather than being stuck in my addiction and just living for my addiction. . . . I realize that I deserve a better life and I love myself. And I have more respect for myself. And the honesty that, just being honest with myself and others, had a major impac^24^t.” (participant)^16^ |  |
|  | “[After] he just took to it like a duck to water really and it has just really improved the quality of his life dramatically, I think he really feels like he’s got a place there and a great purpose … to actually have somewhere where he can feel he has a meaningful role has been important for his self-esteem.” (participant)^1^ |  |
|  | “It’s probably because, like at GONA you get to learn about how it feels to not do that, to not do the drugs and alcohol but to just sit back and just get to really know yourself.” (participant)^21^ |  |
|  | “Gives a better grasp of your identity, [which] makes your experience more positive. Having a sense of who you are [makes] you better equipped to approach situations.” (participant)^15^ |  |
| Encouraging optimism | “My anger has gone down; my anger was up a long time ago. Up until the Our Life program, it’s come down. It helped me, with my anger.” (participant)^17^ | ^5, 16-18^ |
|  | “Positive attitude; more to life than drugs; seeing a whole new world.” (participant)^18^ |  |
|  | “But this program … has given me hope and has allowed me to really think what I wanna do with the rest of my life. And because I was stuck, not stuck, I was I guess you could say rock bottom, you know going home couldn’t get me out of that rock bottom that I was in. But since coming here it’s given me, like I don’t know the word I should use, like...there’s a horizon waiting for me.” (participant)^5^ |  |
|  | “After the retreat I felt like a brick was lifted off of my shoulders and I was just feeling free.” (participant)^16^ |  |
| Reducing carer stress | “My family, they tell me ‘I know you’re still drinking mum, but at least you’re not on the streets, we don’t have to worry about you. We know where we can find you and you know… I wonder how many times …they think that it’s me that’s dead out there when they find a Native woman.” (participant)^5^ | ^1, 4-5, 9^ |
|  | “I couldn’t take him anywhere, we used to up until Christmas … I used to teach night classes and I used to bring him, I actually enrolled him, he didn’t do anything he wasn’t able to, I needed to have him there because I didn’t have anyone to care for him.” (carer)^1^ |  |
|  | “I wasn’t getting enough of a break, and there just didn’t seem to be any way to get a break, it was just putting one foot in front of the other, he was housebound, I was largely housebound, um, yeah, if I did get a break, I was often so tired that I’d just go into my room and sleep, so yeah, although I was very grateful for it, it wasn’t enough.” (participant)^1^ |  |
|  | “I always think that when he’s home (alone) I always think he might fall down or walk the wrong way.” (carer)^1^ |  |
|  | “It’s given me a life back … Well I feel a lot freer, a lot more rested, I have more options, more time to do stuff, um as we are getting more into the routine of it, I guess I’m counting on it more.” (carer)^1^ |  |
|  | “We as family panic about every little thing. It was much easier when we knew [NP] was going to be near and the on-call nursing was available.” (carer)^1^ |  |
| **Threats to long-term program viability** | |  |
| Apprehensive about losing programs | “I think the hurdles for running the house is looking for the right people to work in there, and that’s an interesting process in itself, and you’re always going to have those problems in Alice Springs, of getting the right personalities. I know programs that have fallen over because certain people have left and the passion has left with them.” (staff)^1^ | ^1, 3-4, 14^ |
|  | “The biggest challenge now is getting us up the next 20 percent. And that’s going to take different approaches, whether that’s different techniques of screening or making better use of the navigator program to do outreach [ . . . ]. Unless we do something different, we’re going to stay where we are.” (Staff)^24^ |  |
| Undermining community trust | “That would be like off brand…like that would not be comfortable.” (participant)^2^ | ^2,9, 13, 19-20, 23^ |
| Waning Interest | “You know I’ve heard this so many times, all the good stuff is jacked right up. And all the junk that they buy, it’s no choice you know because it’s cheaper, it’s cost efﬁcient for them. All the good stuff should be cheaper [.] Not only that, but I think this should come from the factories themselves you know. They should involve the government, the factory makers, you know decide if you want everybody to be diabetic in 100 years [.] That’s my main concern, you guys need to step up a few more steps to push for this prevention that we’re doing.” (community member)^11^ | ^11, 13, 20, 23^ |
